# Differential expression of CD10 in prostate cancer and its clinical implication

**DOI:** 10.1186/1471-2490-7-3

**Published:** 2007-03-02

**Authors:** Marc A Dall'Era, Lawrence D True, Andrew F Siegel, Michael P Porter, Tracy M Sherertz, Alvin Y Liu

**Affiliations:** 1 Department of Urology, University of Washington, Seattle, WA, USA; 2 Department of Pathology, University of Washington, Seattle, WA, USA; 3 Department of Management Science, and Statistics, University of Washington, Seattle, WA, USA

## Abstract

**Background:**

CD10 is a transmembrane metallo-endopeptidase that cleaves and inactivates a variety of peptide growth factors. Loss of CD10 expression is a common, early event in human prostate cancer; however, CD10 positive cancer cells frequently appear in lymph node metastasis. We hypothesize that prostate tumors expressing high levels of CD10 have a more aggressive biology with an early propensity towards lymph node metastasis.

**Methods:**

Eighty-seven patients, 53 with and 34 without pathologically organ confined prostate cancer at the time of radical prostatectomy (RP), were used for the study. Fourteen patients with lymph node metastasis found at the time of surgery were identified and included in this study. Serial sections from available frozen tumor specimens in OCT were processed for CD10 immunohistochemistry. Cancer glands were graded for the presence and intensity of CD10 staining, and overall percentage of glands staining positive was estimated. Clinical characteristics including pre- and post-operative PSA and Gleason score were obtained. A similar study as a control for the statistical analysis was performed with CD13 staining. For statistical analysis, strong staining was defined as > 20% positivity based on the observed maximum separation of the cumulative distributions.

**Results:**

CD10 expression significantly correlated with Gleason grade, tumor stage, and with pre-operative serum PSA. Seventy percent of RP specimens from patients with node metastasis showed strong staining for CD10, compared to 30% in the entire cohort (OR = 3.4, 95% CI: 1.08–10.75, P = 0.019). Increased staining for CD10 was associated with PSA recurrence after RP. CD13 staining did not correlate significantly with any of these same clinical parameters.

**Conclusion:**

These results suggest that the expression of CD10 by prostate cancer corresponds to a more aggressive phenotype with a higher malignant potential, described histologically by the Gleason score. CD10 offers potential clinical utility for stratifying prostate cancer to predict biological behavior of the tumor.

## Background

The cluster designation (CD) antigens are cell surface molecules first defined on human leukocytes and later found to be expressed by a variety of human cell types in both normal and pathologic states. The human prostate has been CD immunophenotyped and differences in the expression of several CD molecules were seen between cancer and normal prostate tissue [1]. Among these is the common acute lymphocytic leukemia antigen (CALLA) CD10. CD10 is a 100-kDa transmembrane glycoprotein, also known as neutral endopeptidase (NEP), membrane metallo-endopeptidase (MME), or enkephalinase, involved in the cleavage and inactivation of certain peptide hormones important for signal transduction including the enkephalins, bombesin, and substance P. The biological function of these potential CD10 substrates in the prostate is unknown. Prostate stromal fibromuscular cells, however, express proenkephalin, which may be a substrate for CD10 within the prostate [2]. CD10 is strongly expressed by normal prostatic luminal epithelial cells and is a normal component of human prostasomes [3].

Loss of CD10 expression by metastatic prostate cancer was first reported to contribute to androgen independent tumor growth [4]. In addition to prostate cancer, CD10 expression has been examined in other epithelial cancers including kidney, breast, lung, and skin. CD10 expression is greater in more advanced primary melanomas and is upregulated in metastatic lesions compared with the corresponding primary tumors [5,6]. Normal tissue and well differentiated tumors of the colon and stomach express CD10 while poorly differentiated tumors from these sites show decreased expression [7]. Lung and kidney tumors show a similar pattern of decreased expression compared to the normal parenchyma [8], while hepatocellular and thyroid carcinoma express high levels [9,10]. It may be gathered that CD10 function likely varies by tissue type and disease state.

A high percentage of prostate tumors show an early loss of CD10 expression [1,11]. Tawfic *et al*. [12] reported an absence of CD10 expression by Gleason grade 2 and 3 tumors however noted high expression of cytoplasmic and membranous CD10 in high Gleason tumors. High Gleason grade has been well established as a marker of aggressive biological behavior and is one of the best predictors of patient outcome from prostate cancer that we have available today. While primary tumors show a range of CD10 expression, lymph node metastases show nearly uniform high levels of expression [1]. We postulate that prostate tumors expressing high levels of CD10 correlate with a poor disease outcome. Less than 5% of patients are found to have lymph node metastasis at the time of radical prostatectomy (RP) for clinically localized prostate cancer, but up to 30% of patients will experience biochemical failure (rising PSA) or gross disease recurrence after surgery. These findings may be due to significant clinical under-staging and suggests that many sub-clinical metastases are not diagnosed at the time of surgery for presumed localized disease. Improved prediction of biologically aggressive tumors with a high propensity towards early metastasis would allow for either more aggressive or more appropriate primary treatment. We describe here our finding that CD10-positive tumors are of a more aggressive cancer type predictive of lymph node metastasis and biochemical recurrence after primary therapy. Osman *et al*. [13] have earlier reported a study on prostate cancer CD10 expression. Although our immunohistochemistry data are similar in that a majority of tumors are CD10-negative and CD10^+ ^cancer cells tend to be found in higher Gleason grades, our findings regarding PSA recurrence after RP are at odds. We will discuss possible explanations for our disparate findings in this paper as well.

## Methods

### 1. CD immunohistochemistry

This study was carried out under approval by the University of Washington IRB for the research use of excess tissue from surgeries. Frozen blocks of tumor tissue in OCT were retrieved from the tumor bank in the Department of Urology, and multiple 5-μm serial sections were cut from each specimen and fixed in cold acetone for CD10 immunohistochemistry. Details of this procedure have been previously described [1]. Each specimen was assigned a numeric code with a letter code indicating the site origin of the block (right apex, left mid, etc.). Cancer glands were scored for the presence of CD10 staining, and overall percentage of glands staining positive in the different Gleason component patterns was scored by a single pathologist (LDT). Since benign glands stain positive for CD10, this provided a reliable gauge for positive cancer staining. An isotype control was also done as described [1]. Eighty-seven patients, 53 with and 34 without pathologically organ-confined prostate cancer at the time of RP were identified. The CD10 results of these specimens were reported in ref. 1, and a formal statistical analysis relating CD10 staining and clinicopathological characteristics is presented here. In scoring, each tumor in the particular section examined was characterized as containing what percentage (0 to 100%) of Gleason pattern 3, pattern 4 or pattern 5; then within each pattern, the percent positive for CD10 staining. Fourteen patients with lymph node metastasis found at the time of RP were identified and included in this study. Clinical characteristics were obtained from the medical records.

To further test our methods for estimating percent CD10 staining by specific Gleason pattern for correlation with clinical parameters we elected to perform an identical analysis with a separate CD antigen. Another cohort of 66 patients was scored for CD13 immunohistochemistry and subjected to the same statistical analysis described below. The cancer glands were similarly characterized, and correlation between staining and the same disease parameters was investigated. CD13 was chosen, because it, like CD10, is a cell surface peptide-processing enzyme (ANPEP, aminopeptidase N) expressed by luminal cells, and a majority of cancer glands were also found not to express CD13. Like CD10, a smaller percentage of cancer glands were positive for CD13. The CD13 cohort was not identical to the CD10 cohort, but there was some overlap of subjects. Because of the limited number of lymph node metastases in our collection, the same node specimens were serially stained for CD10 and CD13. Monoclonal CD10 (clone HI10a) and CD13 (clone WM15) antibodies were obtained from BD PharMingen (San Diego, CA). In our hands, these antibodies worked well for frozen sections [1]. Antibodies were used at a concentration of 4 ng/μl. Immunolocalization was done by an indirect avidin-biotin-peroxidase method as described previously [1]. In both these investigations, tissue microarray was not used because of inherent sampling problems with such arrays as only a small portion of any tumor is represented. This would make correlation with clinical outcome difficult as we previously pointed out [1]. While the sections used here were more representative, cancer foci in areas not captured by the sections were nevertheless missed.

### 2. Statistical analysis

Standard statistical summaries and procedures were used for visualization and significance testing of associations with CD10, and with CD13, including scatterplots with Pearson product-moment and Spearman nonparametric correlation measures and tests, cumulative distribution functions with Kolmogorov-Smirnov and Wilcoxon nonparametric tests, and 1-way ANOVA. Nonparametric methods were included due to non-normal distributions and the presence of ordinal variables. For calculation of odds ratios for lymph node metastasis and for estimating PSA free survival, strong staining was defined as > 20% positivity based on the observed maximum separation of the cumulative distributions. PSA free survival was calculated using the Kaplan-Meier method and survival curves were compared using the log-rank test. Only patients with pathologically localized (pT2-T3N0) disease with an undetectable PSA after RP were included in the survival analysis. Patients with lymph node positive disease and patients who received adjuvant therapy were excluded. PSA recurrence was defined as any value ≥ 0.2 ng/ml after an undetectable PSA after RP.

### 3. Gene expression analysis

DNA array analysis of cancer cell lines [14] and sorted prostate cell populations [15] was done with Affymetrix HG-U133 GeneChips. CD26 was used to sort luminal cells, CD104 to sort basal cells, and CD49a to sort stromal fibromuscular cells. In cell sorting, prostatectomy tissue samples free of cancer were digested with collagenase and the resultant cells were partitioned on Percoll density gradients into epithelial and stromal fractions. Magnetic cell sorting (MACS) was used to select the targeted cell populations after labeling with CD antibodies conjugated to R-phycoerythrin (PE). The array datasets were comprised of 2 replicates for the cell lines and 5 replicates for the sorted cells. In addition to the cell types mentioned, datasets were obtained for benign tissue (NP), tumor tissue (CaP), sorted cancer cells from a primary tumor (CD26+ CaP), sorted endothelial cells (CD31) [15], putative epithelial stem cells (CDw338/antibody clone 5D3). A description of the sorted cancer cells and 5D3 cells will be reported elsewhere. Data analysis was done with GeneSpring software.

## Results

### 1. Expression pattern of transmembrane peptidases in prostate cancer

**Figure 1:**
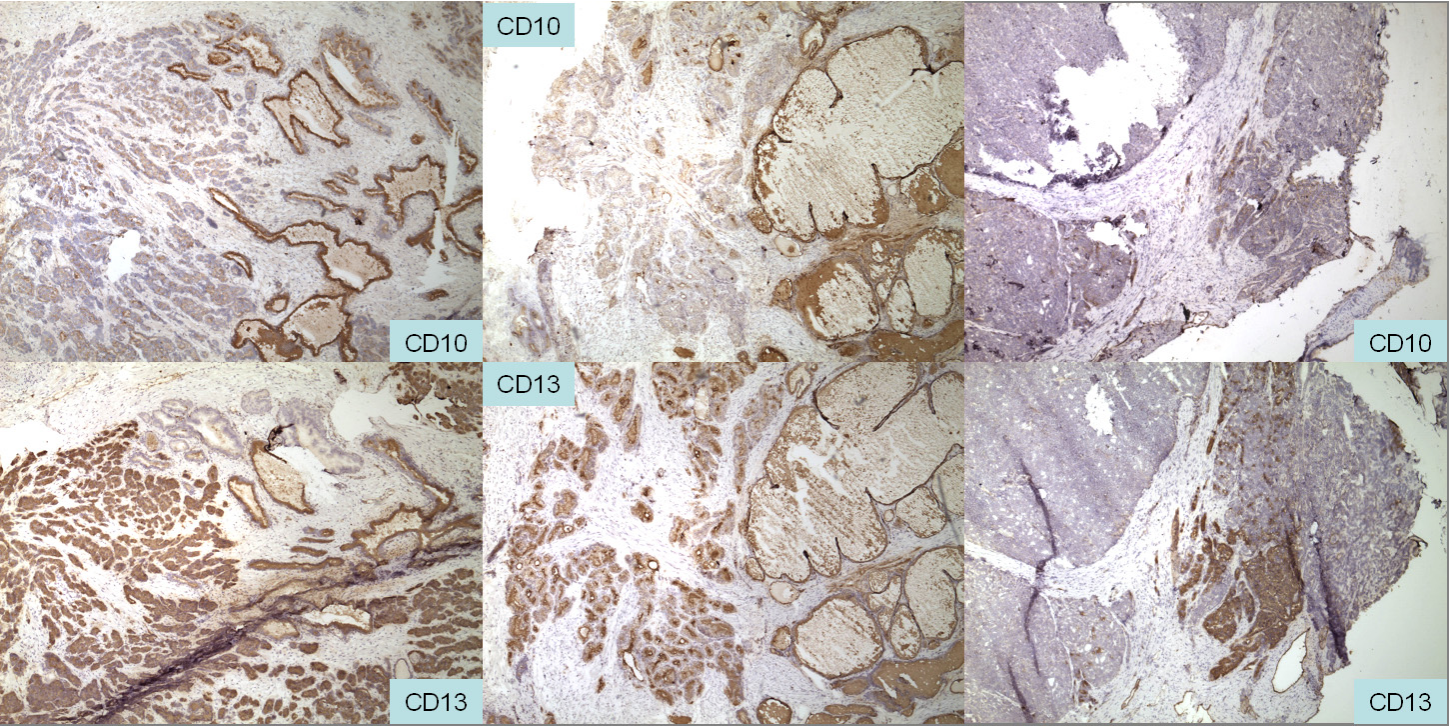
**CD10 and CD13 prostate cancer cell types**. Positive staining is indicated by the brown chromogen deposit. Non-cancer glands are generally positive for both CD10 and CD13. In the left and middle cases the tumors consist of small crowded glands in the left half of the field. Most of the cancer glands are CD10^- ^and CD13^+^, although a few isolated CD10^+ ^cancer glands can be seen. In the right case, a subpopulation of CD13^+ ^cancer cells are present in the larger population of CD10^-^/CD13^- ^cancer cells. This tumor shows no glandular morphology. Magnification is 20×.

**Figure 2:**
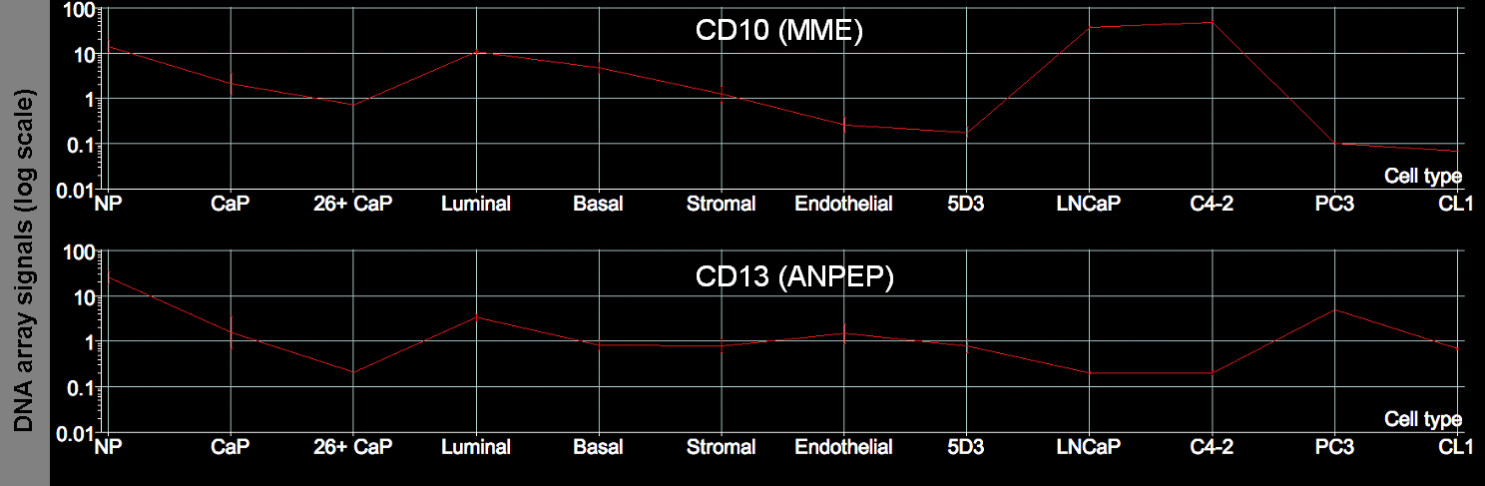
**Gene expression profiles**. Expression for CD10 and CD13 is quantified by DNA array signals on the *y*-axis (in log scale). Most of the published raw data are available from our website. On the *x*-axis are listed the biospecimens analyzed: NP, CaP tissue; sorted cancer, luminal, basal, stromal, endothelial and 5D3 cells; LNCaP, C4-2, PC3 and CL1 prostate cancer cell lines. The expression levels of these genes in the cancer cell lines are in agreement with other experimental results. Note the decrease in CD10 and CD13 between sorted CD26^+ ^cancer cells and normal luminal cells. This is also evident in the NP *vs*. CaP comparison.

Both CD10 and CD13 are expressed by luminal cells of the prostatic epithelium but their expression in cancer is heterogeneous [1]. Examples of all four possible CD10/CD13 cancer cell types – CD10^-^/CD13^-^, CD10^-^/CD13^+^, CD10^+^/CD13^-^, CD10^+^/CD13^+ ^– were detected in our cohort. Normal epithelium present in the specimens showed the characteristic strong CD10 and CD13 expression at the luminal plasma membrane. Fig. 1 shows frozen section examples of tumors stained serially for CD10 and CD13. In all three cases, the cancer glands were mostly negative for CD10. In two cases, the cancer glands were positive for CD13, and a small subpopulation of CD13^+ ^tumor cells was present in one case. These examples showed that CD13^+ ^cancer cells could be found in tumors with either a glandular (Gleason 3) or non-glandular (Gleason 4) morphology. Overall, the predominant cancer cell phenotype was CD10^-^/CD13^-^, and this was validated by DNA array analysis data shown in Fig. 2. As can be seen, the expression pattern was similar for CD13 and CD10 (except in the cancer cell lines) with both CD10 and CD13 expression down-regulated in cancer specimens. For the cell lines, the expression profiles of these two genes were in agreement with the reported flow cytometry and immunocytochemistry data [11,16]. The data also showed that expression of any particular gene was variable in cancer; one cancer cell type may express it while another one may not. Their differential expression in cell lines may reflect the situation in tumors, where either positive or negative cancer cell types are found.

### 2. Correlation of CD10 expression with cancer grade and stage

**Figure 3:**
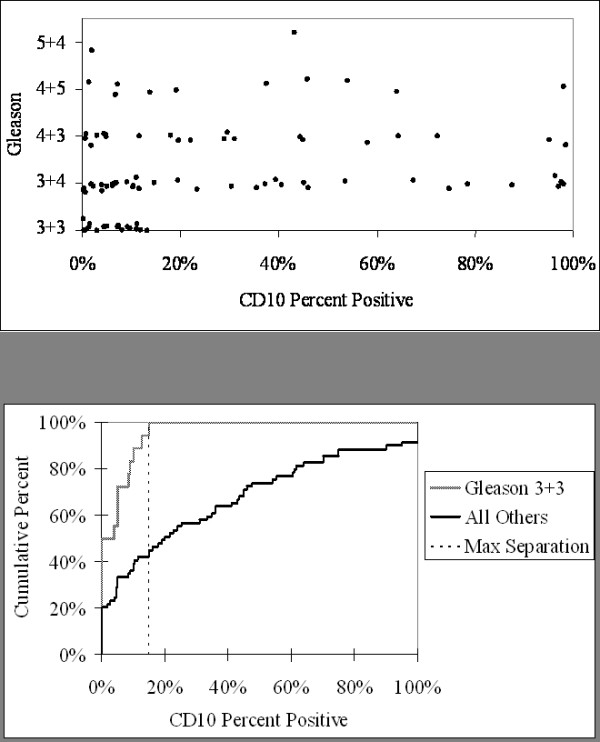
**Percent CD10 positive and Gleason score**. Shown are the scatterplot (top) and cumulative distributions (bottom) of percentage of tumor CD10 positivity and Gleason score. Differences in CD10 cancer expression are significant when Gleason scores are sorted into 3+3 and > 3+3.

**Figure 4:**
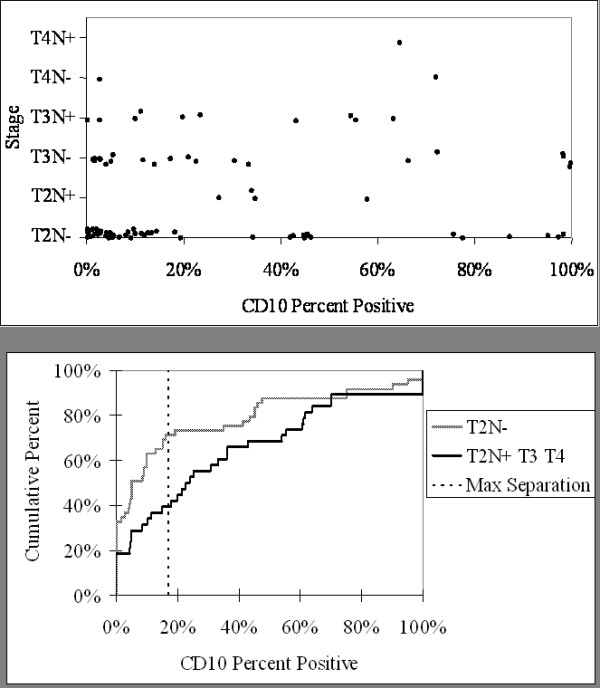
**Percent CD10 positive and stage**. Shown are the scatterplot (left) and cumulative distributions (bottom) of percentage of tumor CD10 positivity and clinical stage. Differences in CD10 expression are significant when stages are sorted into T2N- and T2N+/T3/T4.

**Figure 5:**
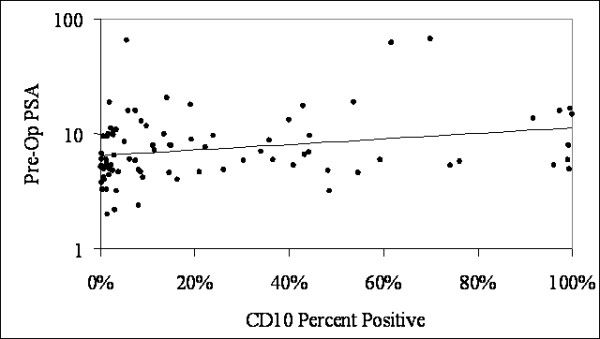
**Percent CD10 positive and pre-operative PSA**. The scatterplot shows the relationship between the percentage of CD10 positivity and serum PSA level before surgery.

In the CD10 cohort, a trend was seen that in tumors with predominantly pattern 3 cancer (Gleason score 3+3 = 6) the percentage of positive CD10 staining was less than 5–10%. Higher percentages were found in tumors with patterns 4 or 5, although some pattern 3 tumors were also positive [1]. Correlations between CD10 percentage and Gleason score, clinical stage and pre-operative serum PSA level are summarized in Table 1 and are as follows. The first scatter plot (Fig. 3) shows the relationship between percent positive and Gleason score with jitter added to allow separation of multiple observations with identical data values for viewing purposes. The result was pooled based on relative similarity at advanced grades, combining all cases more advanced than 3+3 into a single group (n = 69) and compared to that of 3+3 (n = 18). Clinically, there is a clear behavioral difference between Gleason pattern 3 and pattern 4/5 tumors. The result, shown as cumulative distributions, was very highly statistically significant (Wilcoxon *Z *= 3.645, P = 0.0003; Kolmogorov-Smirnov *D *= 0.551, P = 0.0003) with higher percent positive associated with advanced Gleason scores. The mean increased from 4.1% to 32%, while the median increased from 2% to 20% for Gleason score 6 and Gleason score 7, 8 and 9 tumors, respectively. The maximum separation of the cumulative distributions, 100%-44.9%, occurred at 15% positive. The result was significant across all Gleason scores (1-way ANOVA *F *= 4.214 with 3 and 83 degrees of freedom, P = 0.00796). The Spearman correlation was ρ = 0.336, P = 0.00148 with n = 87. The second scatter plot (fig. 4) shows the relationship between percent positive and stage. The result was pooled based on the relative sparseness at advanced stages (combining stages T3 and T4), and was separated to compare organ confined (T2N-, n = 49) to non-organ confined stages (T2N+, T3, T4, n = 38) as shown in the cumulative distributions. The differences were statistically significant (Wilcoxon *Z *= 2.316, P = 0.0206; Kolmogorov-Smirnov *D *= 0.320, P = 0.0253; Spearman ρ = 0.234, P = 0.0293 with n = 87), with more advanced stages having significantly higher percent positive staining. The mean increased from 20.6% to 33.5%, while the median increased from 5% to 23.4% for the organ confined and non-organ confined stages, respectively. The maximum separation of the cumulative distributions, 71.4%-39.5%, occurred at 17% positive. The third scatter plot (Fig. 5) shows the relationship between percent positive and pre-operative PSA level (logarithmic scale, n = 84 observations after 3 were omitted due to missing values). There was a statistically significant relationship, with a correlation of 0.262 (P = 0.012), with higher percent positive associated with higher PSA levels. The relationship and its significance were robust to the 3 possible outliers with the highest PSA levels (correlation of 0.241, P = 0.0299, when these were omitted). The Spearman correlations were ρ = 0.300, P = 0.00555 with n = 84, and ρ = 0.279, P = 0.0118 with n = 81 minus the 3 outliers.

**Figure 6:**
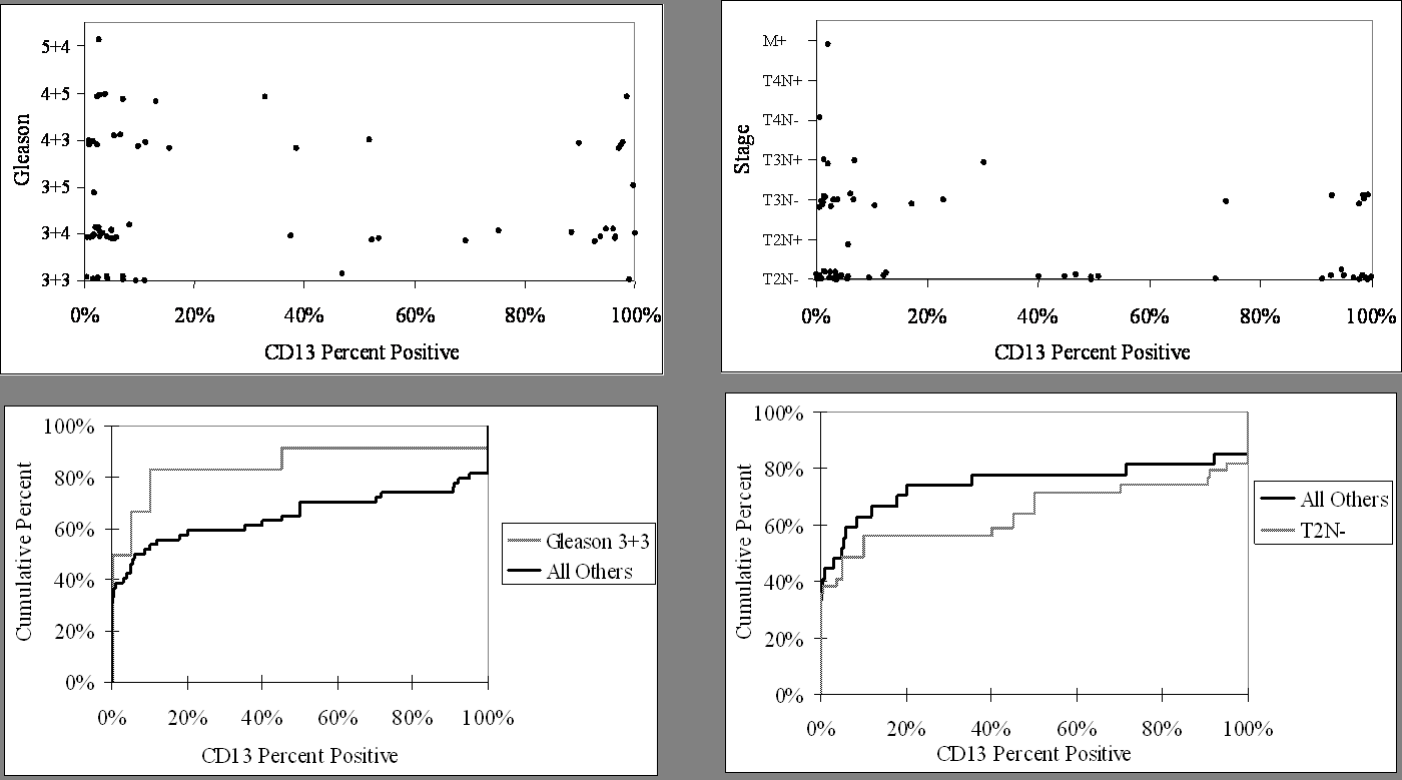
**Statistical analysis of CD13 positivity and disease characteristics**. Shown are the scatter plot (left top) and cumulative distributions (left bottom) of percent CD13 positive and Gleason score, and also those (right top and bottom respectively) for percent positive and stage; differences are not statistically significant.

The second analysis showed no statistically significant correlations between CD13 and the same pathologic and clinical parameters. A scatter plot (Fig. 6) shows the relationship between percent positive and Gleason score. The result was analyzed by combining all cases that were more advanced than 3+3 into a single group (n = 54) and compared to that of 3+3 (n = 12). The resulting cumulative distributions showed that the relationship was not statistically significant (Wilcoxon *Z *= 1.439, P = 0.075; Kolmogorov-Smirnov *D *= 0.296, P = 0.355; Spearman ρ = 0.0700, P = 0.576 with n = 66). The next scatter plot shows the relationship between percent positive and stage. When the result was separated to compare T2N- (n = 39) to the more advanced stages (n = 27), as shown in the cumulative distributions, the differences were again not statistically significant (Wilcoxon *Z *= 0.548, P = 0.584; Kolmogorov-Smirnov *D *= 0.214, P = 0.460; Spearman ρ = -0.111, P = 0.375 with n = 66).

### 3. CD10 and lymph node involvement

**Figure 7:**
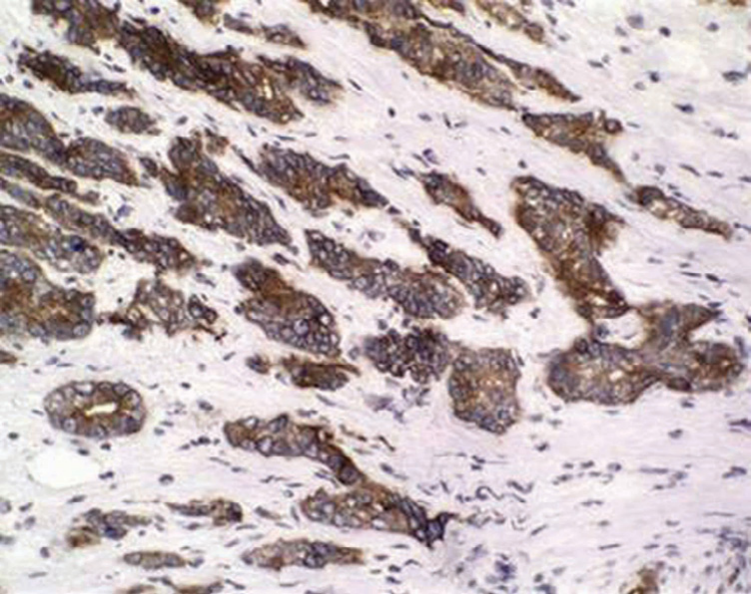
**CD10 immunohistochemistry of prostate cancer lymph node metastasis**. Positive staining is indicated by the brown chromogen deposit. Magnification is 40×.

Nearly all lymph node metastases available in our collection (8/9) displayed strong staining for CD10 (Fig. 7). Seventy percent of RP specimens from patients with node metastasis showed strong staining (> 20%) for CD10, compared to 30% in the entire cohort (OR = 3.4, 95% CI: 1.08–10.75, P = 0.019). Thus, patients with tumors staining heavily for CD10 were more likely to harbor lymph node metastasis at the time of RP. Tumor cell lines derived from node metastases, LNCaP, C4-2 (Fig. 2) and xenograft LuCaP 35 [16], are CD10^+^.

### 4. CD10 and PSA recurrence

**Figure 8:**
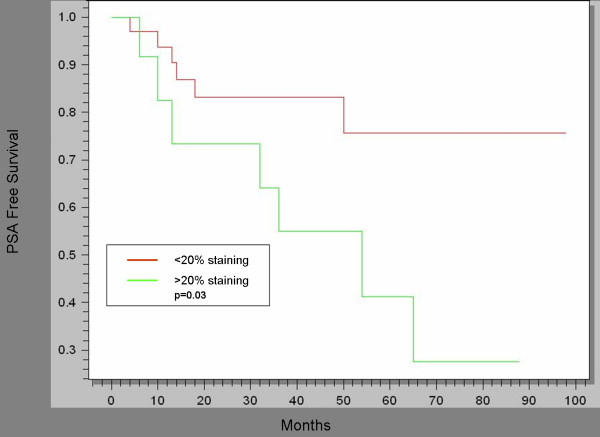
**Percent CD10 positive and PSA recurrence**. Kaplan-Meier analysis shows significantly lower PSA free survival for cohort with > 20% staining for CD10.

Thirteen patients in our cohort with a mean follow-up of 29 months experienced PSA recurrence after treatment while 32 remained disease free. Figure 8 depicts the Kaplan-Meier PSA free survival stratified by percent positive staining for CD10. Patients with > 20% staining for CD10 showed significantly worse PSA free survival after RP (P = 0.03).

## Discussion

Prostate cancer is a heterogeneous disease with different clinical presentations, responses to therapy, and long-term outcomes. One of the more difficult challenges in treating this disease involves identifying and distinguishing aggressive tumors from those likely to remain indolent with little detriment to the patient. Recent advances in high-throughput genomic and proteomic analysis of prostate cancer have begun to describe the biological differences of these tumor types at the molecular level. Prostate cancer primary tumors show heterogeneous expression of CD10 with early loss of expression by many tumors [11]. Most lymph node metastases, however, strongly express the antigen suggesting that CD10 expression may be a marker for or involved in the pathogenesis of metastatic prostate cancer. In our cohort of men, high CD10 expression correlated with advanced Gleason score, and thus with more aggressive tumors with poor pathological and biochemical outcomes. We found that patients with primary tumors expressing high levels of CD10 were more likely to harbor lymph node metastasis and experience PSA recurrence after RP. Comparative array analysis among Gleason pattern 3, pattern 4 and pattern 5 tumors microdissected by laser-capture showed increased CD10 expression in the higher patterns [17]; a trend also reported by the study of Osman *et al*. [13].

In contrast to our results, Osman et al. propose that loss of CD10 expression is associated with an unfavorable patient outcome. This group found that complete loss of CD10 expression was associated with PSA recurrence after RP. Since a majority of tumors are CD10-negative (found in both of our cohorts), their conclusion would suggest a higher failure rate than is observed clinically, and would contradict the well established risk for disease recurrence by Gleason score (lower Gleason score tumors tend to be CD10-negative). Our analysis was different and showed decreased PSA free survival after RP for men with high staining for CD10 in their primary tumor. CD10 expression patterns in the Osman study were not reported by specific Gleason pattern within individual tumors which makes our studies difficult to compare. This study also found a significant association between heterogeneous expression of CD10 and African American race. The majority of cases in the Osman study were African American while our cohort consisted primarily of Caucasian men.

Sampling variations may account for some of the observed differences between our studies. Statistical analysis for correlation of markers with disease outcome is affected by sampling as a tissue section for immunohistochemistry only captures a portion of the tumor in the prostate (see the tumor CD13 heterogeneity in Fig. 1). This is more pronounced when using tissue microarrays [11], unless individual Gleason components of every case are systematically arrayed (which is not usually done).

With our methods, each tumor was analyzed with respect to CD10 staining by the individual component Gleason patterns. We did not observe an absolute trend in staining with all pattern 3 s negative and all patterns 4 s and 5 s positive [1]. Such heterogeneity may make correlations between marker and outcome difficult if not taken into account. We performed a second analysis with another marker, CD13, to test our methods and statistical analysis. Significantly, prostate tumors showed similar heterogeneity in CD10 and CD13 staining but we found no correlation between CD13 staining and clinical parameters. More importantly, there was no increase in CD13 reactivity in the node metastasis specimens. In biomarker analysis, we think it is crucial to analyze expression of candidates (especially those that show differential expression in tumors) in both primary cancer and node metastasis. In contrast to other studies, our analysis was performed on frozen tissue specimens. We have performed IHC for CD10 on adjacent formalin fixed paraffin embedded tissues and have observed similar results.

Albrecht *et al*. [19] have reported a shift from membrane bound to cytoplasmic localization for CD10 in high-grade prostate cancer cells with a loss of CD10 expression in areas of high proliferative activity as indicated by Ki67 immunohistochemistry (n = 24, with perhaps around 7 containing CD10^+ ^cancer cells based on their frequency). It is unclear, however, if individual CD10^+ ^*vs*. CD10^- ^cancer cells were scored for Ki67 (by double labeling for example), and there was no statistical analysis of their data. Given the generally low Ki67 reactivity in prostate cancer [20] we are not sure how to interpret these data regarding lower CD10 expression in areas of apparent high proliferative activity. Furthermore, the authors report no Ki67 staining for lymph node metastases where a majority of cancer cells are CD10-positive.

While cancer cells in lymph nodes were found to be CD10-positive (though not all), cancer cells in other metastases were not (unpublished data). Both LNCaP and LuCaP 35, derived from lymph node metastases, are CD10^+^, PC3 and a more malignant LNCaP derivative, CL1, are CD13^+ ^[11] (Fig. 2). The presence of CD13 in PC3 (established from a bone metastasis) and CL1 (which can metastasize to multiple organs) cell lines might indicate its role in metastasis to other organ sites given that CD13 has been shown to function in metastasis [21,22]. It is possible then that the function of CD10 is employed primarily in cancer spread to lymph nodes. A molecular explanation for the functioning of CD10, since both normal luminal cells and cancer cells express it, may come from our recent analysis that showed association of CD10 with Hsp27 (and others) in C4-2 and LNCaP cells [23]. This association is cancer-specific as CD10 and Hsp27 are expressed by different cell types in benign tissue (CD10 by luminal and Hsp27 by basal). Whether this association is also found in the CD10-positive tumors is difficult to answer because the technique at present is not adequate for the generally small amounts of available clinical material. Prostate cancer expression of Hsp27 has been linked to poor outcome by Cornford *et al*. [24] and therefore, cancer expression of either CD10 or Hsp27 appears to be both associated with a poor prognosis.

If our current findings are verified with another larger cohort with long-term follow-up, CD10 might offer a potential clinical utility for stratifying prostate cancer in an attempt to predict biological behavior of the tumor. An immunohistochemistry-based test can be used in the clinical setting to identify CD10-positive tumors on prostate needle biopsies, which may warrant more aggressive initial therapy or closer surveillance post-operatively. A number of drugs against CD10 are available and potential targeted therapies could be formulated based on these drugs, including monoclonal antibody mediated-delivery of chemotherapy.

## Conclusion

CD10 expression by prostate cancer correlates with Gleason score and other clinical predictors of aggressive disease, notably nodal involvement. The role of CD10 in the pathobiology of early, hormone sensitive prostate cancer remains to be determined.

## List of Abbreviations

RP = radical prostatectomy

OCT = optimal cutting temperature compound

PSA = prostate specific antigen

## Competing interests

The author(s) declare that they have no competing interests.

## Authors' contributions

**MD **performed the IHC, data acquisition, and contributed to study design and manuscript preparation

**LT **performed IHC analysis and interpretation

**MP **and **AS **performed statistical analysis

**TS **performed tissue handling and slide preparation

**AL **performed IHC and contributed to study design and manuscript preparation

All authors have read and approve of the final manuscript.

## Pre-publication history

The pre-publication history for this paper can be accessed here:



## Figures and Tables

**Table 1:** Correlation between clinicopathologic characteristics and increased percent positive staining for CD10.

	**n**	**Spearman Coefficient**	**P value**
	
**Increasing Pre-treatment PSA**	81	0.3	0.006
**Stage ≥ pT3**	38	0.234	0.03
**Gleason ≥ 7**	69	0.336	0.001
